# A Robotic Approach to Understanding the Role and the Mechanism of Vicarious Trial-And-Error in a T-Maze Task

**DOI:** 10.1371/journal.pone.0102708

**Published:** 2014-07-22

**Authors:** Eiko Matsuda, Julien Hubert, Takashi Ikegami

**Affiliations:** 1 Department of Arts and Sciences, The University of Tokyo, Tokyo, Japan; 2 School of Informatics and Engineering, University of Sussex, Brighton, United Kingdom; 3 Japan Society for the Promotion of Science, Tokyo, Japan; Centre national de la recherche scientifique, France

## Abstract

Vicarious trial-and-error (VTE) is a behavior observed in rat experiments that seems to suggest self-conflict. This behavior is seen mainly when the rats are uncertain about making a decision. The presence of VTE is regarded as an indicator of a deliberative decision-making process, that is, searching, predicting, and evaluating outcomes. This process is slower than automated decision-making processes, such as reflex or habituation, but it allows for flexible and ongoing control of behavior. In this study, we propose for the first time a robotic model of VTE to see if VTE can emerge just from a body-environment interaction and to show the underlying mechanism responsible for the observation of VTE and the advantages provided by it. We tried several robots with different parameters, and we have found that they showed three different types of VTE: high numbers of VTE at the beginning of learning, decreasing numbers afterward (similar VTE pattern to experiments with rats), low during the whole learning period, and high numbers all the time. Therefore, we were able to reproduce the phenomenon of VTE in a model robot using only a simple dynamical neural network with Hebbian learning, which suggests that VTE is an emergent property of a plastic and embodied neural network. From a comparison of the three types of VTE, we demonstrated that 1) VTE is associated with chaotic activity of neurons in our model and 2) VTE-showing robots were robust to environmental perturbations. We suggest that the instability of neuronal activity found in VTE allows ongoing learning to rebuild its strategy continuously, which creates robust behavior. Based on these results, we suggest that VTE is caused by a similar mechanism in biology and leads to robust decision making in an analogous way.

## Introduction

In a study with rats, Tolman [1] observed that they seemingly hesitated when they had to choose between one of two rooms, one of which contained a reward while the other was empty. The only cue differentiating the rooms was the color of the doors. A black door indicated that the room provided a reward, and a white door indicated an empty room. To reach the reward, the rats had to learn the relationship between the color of the door and the presence of the reward. During the learning phase, the rats were seen moving their heads from one door to the other as if they were considering which one to choose, which was referred to by Tolman as a conflict-like behavior called vicarious trial-and-error (VTE). In his experiments, Tolman noticed that the number of VTE events (i.e., the number of times that the rat shook its head during one trial) increased at the onset of the learning phase but started to decrease when the performance was stabilized. Based on this observation, VTE has been connected to learning efficiency.

Following Tolman's observations, other researchers started paying attention to the presence of VTE. Hu and Amsel showed a hippocampal contribution to VTE [2]. Johnson and Redish recorded place cell activity in rats' hippocampus, and they observed VTE when the rats were simulating their next decisions internally before acting [3]. These results led to the hypothesis that VTE reflects deliberative decision making, which is a cognitive process that includes searching, predicting, and evaluating future outcomes [4]. This process is computationally slow compared with automated decision-making processes, such as habituation and reflex. But deliberative decision making allows ongoing control to achieve flexible behavior. One rat experiment [5] supports this hypothesis; the authors observed high VTE when the rats were uncertain and had to think about their decisions in the following three conditions: 1) error trials, rather than correct trials; 2) the next trial after making an error (i.e., potential error trials); and 3) when the rats had to switch their strategy.

VTE-like behavior has also been found in other animals. In a human experiment [6], the participants showed VTE-like behavior when they had to actively, instead of passively, explore a given environment. They performed better with VTE-like behavior in the active condition. Tarsitano et al. [7] found that in a detour task, jumping spiders displayed two phases of action: the inspection phase, when the spiders stopped and inspected possible routes toward a target, and the locomotory phase, when the spiders moved toward a single direction. VTE was observed during the inspection phase. Tarsitano concluded that “one can speculate that it is a small but significant jump to use trial and error when choosing a goal to approach.” However, in animal experiments, it is difficult to observe the neural dynamics, which makes it hard to directly investigate the mechanism behind VTE and its resulting behavior.

There are some theoretical models of VTE; Rossler took up VTE as a sign of private simulation [8], and Ikegami studied it as an example of embodied chaotic itinerancy [9], that is, the itinerant motion of an autonomous robot with chaotic instability. From the Bayesian theory view point, Johnson et al. posited that VTE occurs with changing task demands [10]. Still, those models lack body and environment structure that will spontaneously generate VTE.

In this paper, we tried to understand whether VTE is an emergent property of a physical body moving around in its environment. We also investigated the link between VTE, neuronal dynamics, and the efficiency of VTE toward learning. Our methodology is to make a simplified abstract model of VTE, rather than making a biologically elaborate one. This experiment has its basis in the field of evolutionary robotics, where basic features of living organisms are recreated by simple robotic systems [11], [12]. This is to study the essential logic underlying living systems, such as autonomy, evolvability, and embodiment [11]–[15]. In this paper, we especially focused on embodied properties of living organisms. Embodied cognition is the notion that the nature of the mind is determined by the physical characteristics of the body and the environment, rather than controlled only by the central nervous system [13]–[15]. For instance, Bovet and Pfeifer [16] showed the spontaneous development of coherent behaviors in robotic experiments – reward-seeking behavior for instance – just by moving around the environment with a physical body. In their experiment, the robot utilized the morphological structure of whiskers and the physical properties of various sensors, such as infrared or vision sensors. By learning the relationship between those different types of signals, the robot successfully reached the reward. This experiment suggests the important notion that intelligent behavior can emerge from the simple interaction between the body and the environment.

We therefore composed a simple robotic model of VTE to show the underlying mechanism responsible for the observation of VTE and the advantages provided by it, in terms of learning and in terms of dynamical systems. The model that we used is based on Bovet's T-maze learning robots [16], which we reproduced in computer simulations. The model that we used is similar to the environment used when the rats showed VTE. As a result, we demonstrated analogous VTE patterns to those reported in the experiment with rats, in terms of the temporal change in the number of observations, that is, high at the beginning of learning and lower afterward.

In addition to the similar pattern to rats, we have found other patterns of VTE using different parameters, which we classified into the following three groups: 1) a high number at the beginning of learning and low afterward, 2) a low number during the whole learning period, 3) and a high number all the time. From the comparison of the types of VTE, we demonstrated that VTE is associated with the chaotic activity of neuronal dynamics. Depending on the three types of VTE, we also compared the robots' behavior to evaluate adaptability by changing the environmental conditions. The robots with the low VTE pattern changed their behavior drastically due to those perturbations, even exhibiting 0% success, while those with the high-to-low VTE pattern were robust to the perturbations. We suppose that VTE causes sensory fluctuations, enabling our robot to continuously change the connectivity pattern of the neurons. In other words, VTE allows the robot not to have to follow the same trail in a maze, enabling it to change its neural activity. This allows the robot to learn in an ongoing way by continuously gathering information from the environment, creating robust behavior. This reminds us of the concept of “homeodynamic adaptation” suggested by Iizuka and Di Paolo [17] – an agent-based cognitive model of morphological disruption where internal instability allows behavior that is adaptive to changes in the body.

The organization of this paper is as follows: In the Methods section, we detail the environmental set-up and the neural model. This is followed by the Results section for results and analysis of the behavior of the robots. The Discussion section then focuses on the role of VTE and the mechanism behind it in the light of adaptability.

## Methods

**Figure 1:**
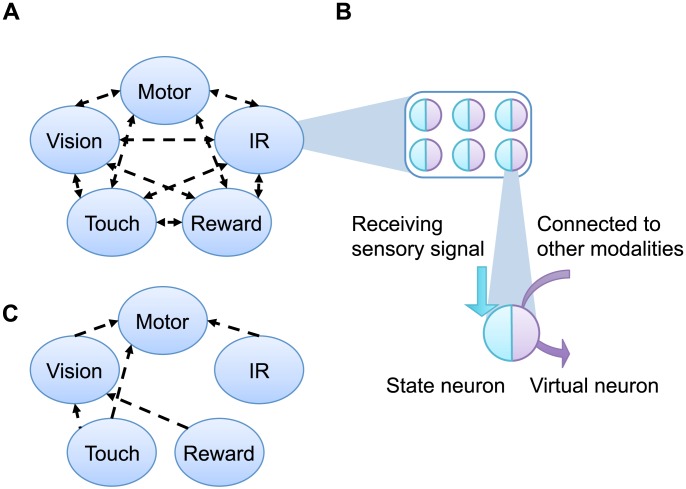
Structure of the neural network. A: Five sub-systems for each sensor or motor modules make up the whole cognitive system of the robots. These modules are fully connected with each other. B: One module is composed of several neurons. Each neuron has two components; one is a state unit, while the other is a virtual unit. C: Modules are minimally connected.

Our work is based on a robot experiment developed by Bovet and Pfeifer [16]. In this model, a robot must reach a goal located on one arm of a T-maze. To do so, a neural network acting as the controller must combine four sensory modules to determine the right motor commands at every instant (Figure 1A). The sensors are labeled tactile, visual, proximity, and reward. These five modules (i.e., the four sensory modules and the motor module) are interconnected and represented by a different neural population within the controller. Synaptic connections are tuned using the Hebbian learning rule, which will be explained in more detail later on. Differently from the original work by Bovet and Pfeifer, our experiments were done in computer simulation. The robot was modeled according to the e-puck robot [18].

**Figure 2:**
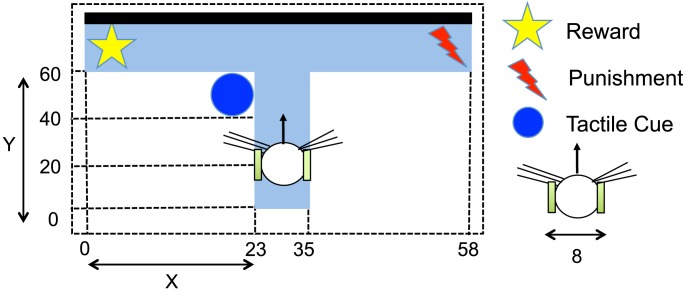
T-maze environment used for the experiment. At the beginning of each trial, the robot was placed on the central arm of the maze. The initial position of the center of the body was originally set to 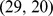. The circle at the choice point represents the tactile cue, the star at one end of the maze indicates reward, and the lightning at the other end of the maze stands for punishment. All the sizes and distances are in centimeters, which is scaled based on actual e-puck robots.

The environment for our study was a T-maze with one central arm and two side ones that is shown in Figure 2 along with its length and detailed arrangement. All the sizes and distances in the simulated environment are in centimeters, which is scaled based on actual e-puck robots [18]. A reward was located at the end of one arm, and a punishment was placed at the end of the opposite one. The task of the robot was to reach the reward by choosing the right arm to follow. The right direction was indicated by a tactile cue at the intersection. The robot had to learn the correlation between the cue and the reward in order to complete the task successfully. Additionally, the robot also had to learn to move around within its environment. The robot was equipped with the following sensors and motors in the simulation environment:

1. **Tactile sensors**: Tactile stimulation came from 32 whiskers attached to the left and right sides of the robot. The length of the whiskers was 20.0 cm (Figure 2). The signals from those whiskers were given as the binary numbers 1 (triggered) or 0 (at rest). The whisker sensors only detected the tactile cue at the corner of the T-maze when the robot was close enough.
2. **Vision sensors**: Visual stimulation reflected the activity of the omnidirectional camera, which returned grayscale values standardized from 0 to 1. This camera was composed of 20 pixels aligned horizontally. Everything in the T-maze was made white or transparent except for the black backside wall (Figure 2). Therefore, only this wall provided a signal of 1 when entering the field of view of the camera. By sensing this black wall, the robot could estimate its location and direction within the arena.
3. **Infrared (IR) proximity sensors**: Six IR proximity sensors were uniformly attached to the front half of the robot's body. These sensors detected the distance from the robot to the walls of the T-maze.
4. **Reward sensitivity**: The reward sensitivity was usually set to 0. It was raised to 1 to signal a reward and lowered to -1 to indicate punishment.
5. **Motors**: The forward velocity of the robot was set to a constant positive value (denoted by 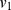). The turning degree was determined by the output of the neural network 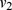, which was standardized between 0 and 1. The vector (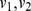) were used to set the left and right wheel velocities as follows:
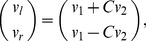
(1)where 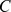 was a constant for converting the standardized value to the actual motor speed. If 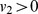, then 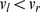, which made the robot turn left, and 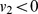 produced a right turn.

All the experiments described in this paper took place in two phases: a familiarization phase and a maze-solving phase. During the familiarization phase, the robot explored the maze with the cue and reward deactivated and with the motor speed fixed to the two variables (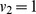 or 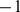, which induced a left and right turn, respectively). When it touched a wall, the robot was returned to its initial position and the motor speed was switched to the other value. This process was repeated 20 times. The purpose of this phase was to give the robot a chance to become familiar with the experimental arena, by learning relationships between the basic modules, that is, the IR, vision, and motor modules. After the familiarization phase, the robot started the maze-solving phase, which consisted of the robot seeking the reward and learning the correlation between the positions of the cue and of the reward, as described above (for more details about the learning processes, see Text S1 and Figure S1–S4 in File S1). The maze-solving phase had 100 trials, where one trial was finished if the robot reached the reward or punishment or was timed out (after 4,000 time steps). The familiarization phase facilitated the maze-solving phase, as the robot had to learn the basic sensor and motor correlations necessary for moving within the maze. The results presented later on only concern the maze-solving phase.

### Neural Network

In this section, we describe how the sensory or motor modules temporally updated their neural states. It is important to note that our neural network is only intended as a behavioral model, rather than as a model of biological neurons. The goal of this simplified model is to focus on a dynamical structure that emerged only from the interaction between body and environment, which we expect will reveal an essential mechanism that causes VTE.

Just for simplification, our description is different from that of the original model [16], but it is still mathematically equivalent to the original version and satisfactory in terms of replication of the model. Each module had a specific number of neurons (tactile 32, vision 20, IR 6, reward 1, motor 1), which were connected to neurons in other modules. The respective neurons were composed of two units: state units and virtual units. The state units were set proportional to sensory signals obtained from each sensors (and motors) and used to tune the Hebbian synaptic weights. The virtual units provided a parallel pathway for sending signals to other modules, which were activated by internal signals sent from other modules (illustrated in Figure 1B). The reason for having the two types of units was to separate the dynamics of Hebbian learning from the internal neural dynamics. The separation into two types of unit was necessary in order to avoid an unwanted positive feedback in which simultaneous firing leads to a strengthening of the connection, which results in a greater likelihood of simultaneous firing in the future. Although the network structure that we used here was specific to the Bovet-Pfeifer model, we think the obtained results do not depend on the detailed architecture of the model. The separation of Hebbian learning from the internal dynamics is the most important step in order to reproduce our result. Both types of units were activated as follows:

1. **State units to compute the Hebbian synaptic weights**: State units 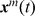 were just set proportional to the sensory signals. For instance, tactile stimuli from the 32 whisker sensors gave sensory values (0 or 1) to the corresponding 32 nodes of 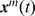. The proportional constants for the light, touch, and reward modules were determined by the genetic algorithm (GA) explained below. That of the IR was fixed to 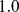. Depending on the state units, the weight matrix from module 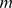 to 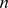 (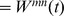) was updated using a modified version of the Hebbian learning rule:
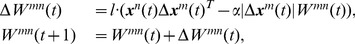
(2)where 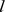 was the learning rate, 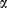 was the forgetting rate, and 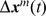 was the difference between the current and delayed state units (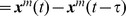). This equation means that the weights from module 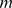 to 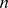 were strengthened when signals of module 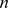 and the signal change of module 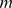 were both high. In other words, the weight matrix encoded associations between sensory inputs, motors, and reward signals. The reason the signal change was taken into account is because it often has meaningful information rather than the state itself, as is explained by [16]. For instance, the motor state could be correlated with optical flow rather than a stable visual image.
2. **Virtual units, to send signals from one module to another**: Depending on the Hebbian synaptic weights computed above, the virtual units 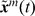 were activated by signals sent from the other modules by the following equation:
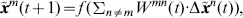
(3)where 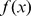 was a sigmoid function 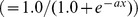 and 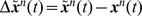, that is, the difference between the virtual units and the state units of the 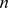 th module. The virtual units did not have any effect on the learning rule, but they sent signals to the other modules through the learned synapses. As an exception, the virtual unit of the reward module (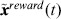) was not subjected to the update by Eq. 3 but constantly set to 1. The virtual units other than the reward module and all the synaptic weights were set to 0 at the beginning of both the familiarization and the maze-solving phase. The virtual unit of the motor module was equivalent to 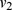 at Equation 1 to update the motor velocity.

The reason that the update function was based on 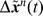 (i.e., the difference between the state and virtual unit) is discussed in (Text S1 and Figure S1–S4 in File S1), and here, we just give a summary. The virtual unit can often be interpreted as an ideal state [19], in the sense that the robot's motion tends to make an actual state closer to the virtual (or ideal) one. In the reward module, the virtual unit was constantly set to 1.0 as explained above, and the robot spontaneously chose actions to make the state unit closer to it, that is, receiving the reward (Text S1 and Figure S3 in File S1). For the IR module, the virtual unit gave rise to wall avoidance behavior, as described in Text S1 and Figure S1 in File S1.

All aspects of the model were updated every 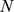 time step, with the exception of the reward states and the robot's velocity, which were updated every time step. 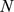 was a parameter between 10 and 30, determined by GA, which is detailed in the next part. The following are the steps that led to the generation of the outputs:

1. Sensory information was transferred to the state units.
2. The Hebbian learning rule was applied on the weight matrix 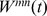 depending on the state units (Equation 2).
3. The activities of the virtual units were updated (Equation 3).
4. Finally, the virtual units of the motor module 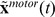 determined 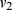 to calculate the motor output (Equation 1).

### Setup of the Genetic Algorithm (GA)

Bovet's robot relied on the following parameters: learning rates 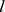 and forgetting rates 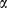 for each directed pair of sensor or motor modules (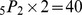; Equation 2), update frequency of the neural network 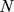, 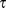 for the delayed states, the constants 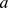 for the sigmoid function, 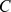 at Equation 1 (for each familiarization / maze-solving phase), the forward velocity 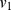 (Equation 1), and the proportional constants for the light, touch, and reward module. As is generally the case with robotic models, only a subset of values within the parameter landscape will provide us with controllers capable of solving the task. Within this subset, small variations of the parameters can produce slight differences in the performance of the robot. As such, in order to make sure that our results are not due to underperforming controllers, we tuned those with GA. To tune these parameters and optimize the performance of the controller, we employed a standard GA [20]. We used a population of 100 individuals to optimize the 49 parameters and used a tournament selection with a single point cross-over operation with a probability of 70% and a 1% mutation rate. We also applied elitism by simply copying the five best individuals to the next generation without applying a mutation. The fitness function 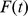 of an individual evaluated at the generation 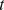 was calculated as:
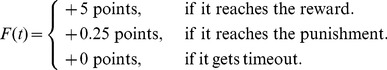
(4)


The fitness rewards were determined experimentally. The trials were repeated 100 times from one fixed initial position, which gave a maximum fitness value of 500.

### Plausible Mechanism to Generate Reward-Seeking Behavior

As was described above, we found several possible parameter sets by GA to maximize the fitness value. Although those different parameter sets might result in various strategies, here we briefly explain the most plausible mechanism to generate the reward-seeking behavior of the robot [16], [21]. For more details, see (Text S1 and Figure S1–S4 in File S1). It seems plausible that the reward-seeking behavior was generated from the following two neuronal paths learned by Hebbian rule:

#### Reward – vision – motor correlation

This path leads the robot to go to the previous reward position. This can support the reward-seeking behavior at the earlier stage of the T-maze task, that is, before acquiring the meaning of the tactile cue.

#### Tactile – motor correlation

This path lets the robot follow the tactile cue to make a correct decision. At the beginning of the task, nothing about this path is learned, but it will gradually be acquired by moving around the environment.

The position of the reward changed every five trials of the T-maze task, so that the trials after switching the reward position could be in conflict because of the two neuronal paths described above. Namely, the first neuronal path would lead the robot to go to the past and wrong reward position. The second path would tell the robot the correct reward position suggested by the tactile cue. Therefore, before maturation of the synapses, this experimental setup could cause a conflict-like behavior, or VTE. However, depending on the parameters, the robot could still solve the maze by different learning procedures.

### Neural Network – Minimally Connected Model

As the task presented in this paper is simple conditional learning, it can be performed using a minimal network topology. In the neural network described above, the sensor and motor modules were fully connected, which could be redundant to solving the task. We hypothesized that VTE could be generated from the redundant connectivity. Therefore, we composed a minimally connected model by omitting redundant connections. To show the relevance of redundancy with VTE, we compared the behavioral difference between the evolved but redundant network and the minimally connected network.

The minimal network we propose did not have redundant connections, but was composed of connections with specific roles to solve the task, that is, “touch–vision,” “IR – motor,” “touch – motor,” and “reward – vision – motor.” Although other combinations could also prompt a successful behavior, we took this network for being the most plausible as a minimal network: The reason for choosing the first two paths are described in the previous subsection (named “Plausible Mechanism to Generate the Reward-Seeking Behavior”), while the roles of the last two are detailed in (Text S1 and Figure S1–S4 in File S1).

## Results

As we have described in the previous section, we examined two types of neural networks in the robot – a fully connected one and a minimally designed one. For the fully connected network, we ran the GA 95 times. Of these, 22 runs produced a network that attained a maximum fitness value (100% success). For the minimally connected network, two out of five runs of GA attained the maximum fitness. Each run of GA was composed of 1,000 generations, and we used robots at the 1,000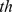 generation for analyses.

All the evolved robots showed the maximum success rate (i.e., 100%), which means that they were successful in the task from the beginning of learning. This seems a bit weird because, even before learning, the robot seemed to know the correct answers. At the very beginning of learning, the robot did not possess any explicit knowledge about the reward and tactile cue so that they solved the maze only by chance. Because the evolved robots showed the maximum success rate, the learning speed did not differ within the 22 evolved robots. But it would be important to note that the evolution speed for the GA, that is, the number of generations to converge on the maximum fitness, differed; the L robots tended to evolve faster than HL / H robots (data not shown).

We first counted the number of VTEs associated with these evolved robots. In his experiment [1], Tolman counted the number of VTEs through the oscillations of the rat's head direction; that is, one VTE was counted if the rat looked at one door and then it looked at the other door. We followed this method for counting the number of VTEs in our robot. In our experiment, since the robot did not possess an independent head from its body, we counted the left and right oscillations of the whole body as VTEs. More precisely, a VTE was granted when the motor output 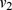 from Equation 1 changed its sign. In order to filter the noisy fluctuation around the turning degree of 0, we counted a movement as a VTE when the sign change of 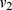 was larger than the threshold range 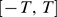, which was set to 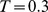. As we discuss later, the threshold 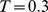 was varied to see the role of VTE.

**Figure 3:**
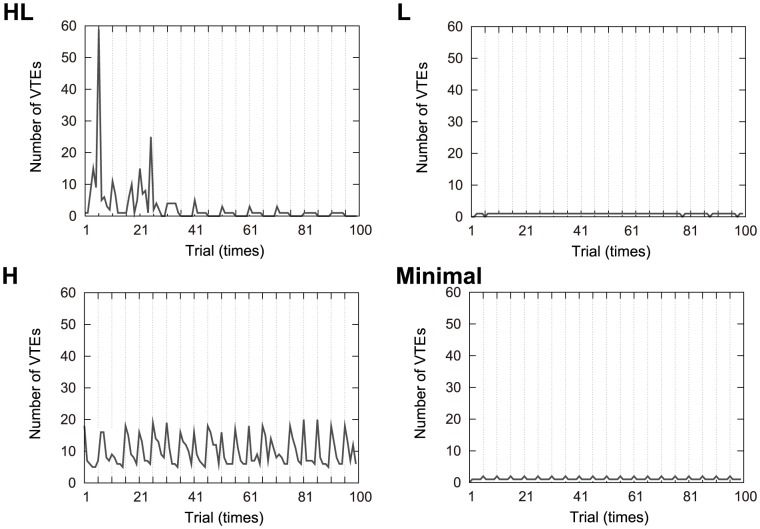
Examples of the number of VTEs shown by individual robots during the 100 trials. The *x* axis indicates the trial numbers, and has auxiliary lines every 5 trials. The *y* axis show the number of VTEs. HL: High to low VTE. L: Low VTE. H: High VTE. Minimal: Minimal model.

**Figure 4:**
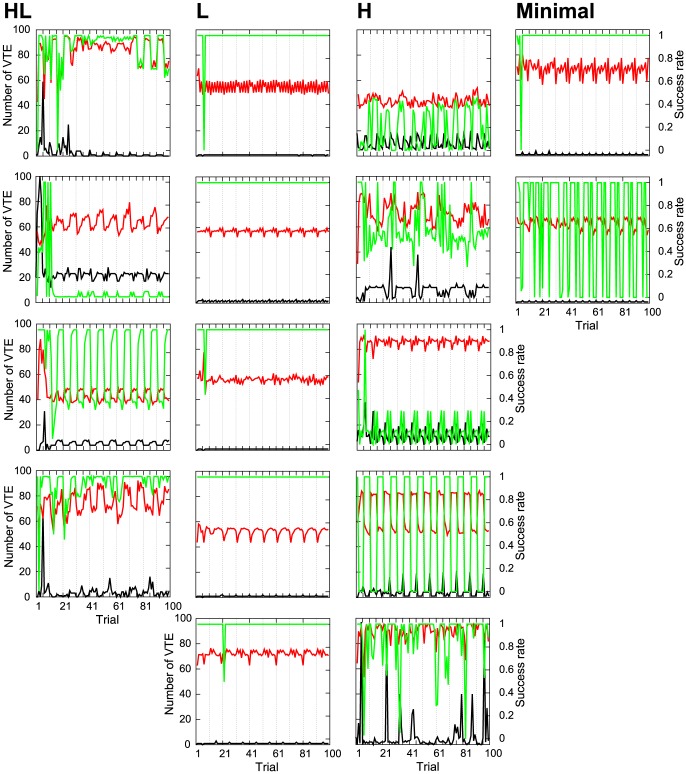
The number of VTEs for the evolved 16 robots and a comparison of the performance with and without learning. Each figure represents the result from an individual robot. The *x* axis denotes trials and has auxiliary lines every 10 trials. The scale of each axis is set to the same value in every figure. Red line: Success rate when the robot ran the trial with learning on. Green line: Success rate when the robot replied the trial with learning off. Black line: Number of VTEs observed in the original condition (i.e., with learning activated and from the original starting point; the same condition as Figure 3) HL: High to low VTE. L: Low VTE. H: High VTE. Minimal: Minimal model.

The evolved robots showed different patterns of VTE that can be classified into three groups. The classification was done by hand, and six out of the 22 evolved robots could not be classified, as they showed exceptional forms of VTE. Therefore, we had 16 robots to be classified. Some examples of VTE dynamics exhibited by individual robots are shown in Figure 3, where the *x* axis denotes trials with auxiliary lines every five trials, and the *y* axis represents the number of VTEs. The results from all the 16 robots are shown in Figure 4 (black lines), which will be shown below in this paper. The three groups are as follows:

### 

#### A high to low number of VTEs (denoted by “HL”)

The number of VTEs increased at the early stages and then gradually decreased after a certain point (Figure 3HL and 4HL). This observation was similar to the experiments with rats [1], [4]. We had four robots out of the 16 that showed this type of VTE. For the robot shown in Figure 3HL, the number of VTEs had its peak at the sixth trial, when the position of the reward first changed. As for the other three evolved individuals in Figure 4, the second one also showed the VTE peak at the sixth trial. The third and the fourth one took the peak at the seventh trial. Therefore, VTE had its peak just after the reward's first switching event, that is, in the most uncertain condition. This peak disappeared later on after the robot had more time to learn the task.

#### Low VTE (denoted by “L”)

The number of VTEs was kept constantly low throughout the 100 trials (Figure 3L and 4L). Five robots exhibited this type of VTE.

#### High VTE (denoted by “H”)

The number of VTEs was kept high (but temporally fluctuating) throughout the 100 trials (Figure 3H and 4H). Five robots had this type of VTE. Two out of the five robots had local maxima in the number of VTEs when the reward position switched, while the other three did not.

The two robots with minimal connectivity (denoted by “Minimal”) were all classified as having low VTE (Figure 3 Minimal). This suggests that the redundant connectivity between the sensor and motor modules was necessary. Figure 4 (black line) shows that there was a continuum between HL and H robots, while the robots of L (and Minimal) seem distinct from those two VTE-showing groups.

Several robots, as shown in Figure 4, exhibited regular changes in the number of VTE; that is, they showed a slightly higher amount of VTE every fifth trial. This may be because the reward position changed every five trials, which affected the robot's behavior.

**Figure 5:**
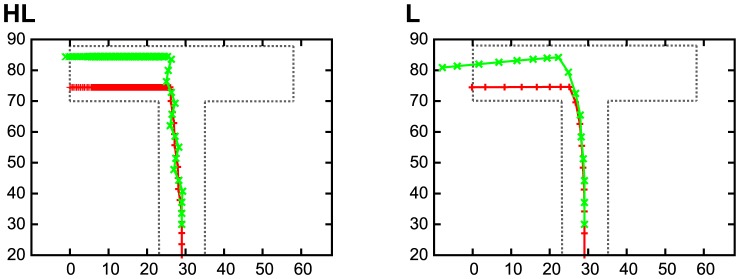
Trajectory of the robot at the sixth trial. The red line indicates the position of the robot, while the green line represents the direction of the angular velocity. HL: High to low VTE. L: Low VTE.

In this experiment, VTE events were measured all along the trajectories of the robot, while several experiments with rats [3] counted the number of VTEs only at the cross point of the maze. However, we consider our way of counting VTEs did not lead to qualitatively different results. Our robots had a fixed forward velocity (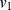 in Equation 1) so that it was not possible that the robot stayed in the vertical arm of the T-maze to exhibit VTE. Indeed, Figure 5 shows some examples of the robot's trajectories, where the red line indicates the position of the robot, and the green line represents the direction of the angular velocity. These figures come from one HL robot and one L robot at the sixth trial, when the HL robot exhibited VTE more frequently than the L robot. The HL robot in this figure hit the back wall of the T-maze, during which the robot changed its head direction often to create VTEs. Namely, the VTEs were generated after the robot sensed the tactile cue. As shown in this example, the VTE appeared to be created in the horizontal arm of the T-maze (i.e., after sensing the tactile cue) because of the fixed forward velocity. It is therefore plausible to say that our way to count VTEs is not qualitatively different from those experiments with rats [3].

Despite the difference in the number of VTEs, each robot showed a perfect success rate (of 100%), which may imply that VTE might not be directly related to the degree of performance in the T-maze task. This seems inconsistent with what is observed in animal experiments [2], [6], where VTE is efficient for learning performance. Still, below we suggest that VTE is efficient for robust learning under environmental perturbation. In the remainder of this paper, we describe a possible mechanism for generating VTE and then evaluate the function of VTE in terms of robustness and dynamic stability. Finally, we discuss the effect of VTE on Hebbian learning.

### The Mechanism behind VTE

**Figure 6:**
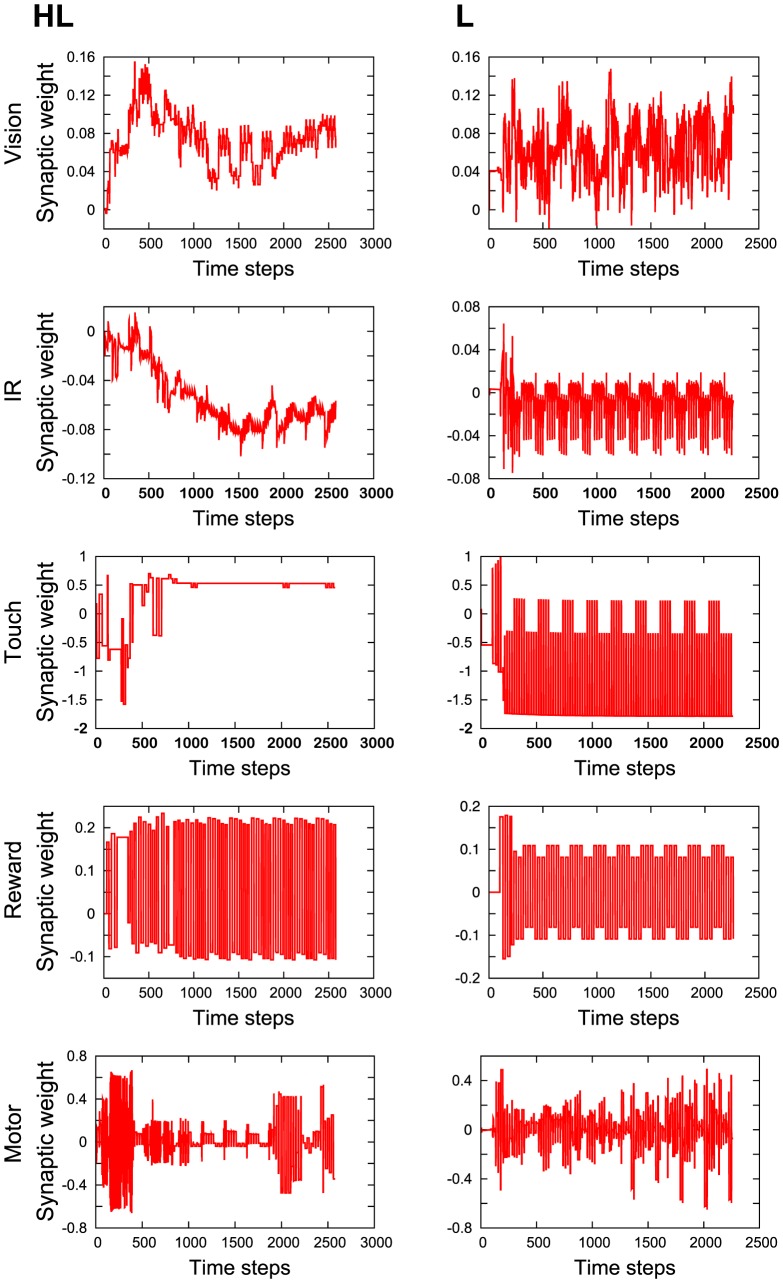
Examples of synaptic weight dynamics of the HL and L during 100 trials. The 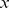 axis denotes time steps, while the *y* axis shows the strength of the synaptic weights. In this figure, synaptic weights belonging to a same module were averaged and shown as one single time series. HL: High to low VTE. L: Low VTE.

In order to understand what differentiates the VTE patterns, we looked at changes in the synaptic weights during learning. Figure 6 presents some examples of the temporal dynamics of the synaptic weights in the HL and L robots. In this figure, synaptic weights belonging to the same module were averaged and shown as one single time series. The entire time series consisted of 100 successive trials, with each trial being comprised of roughly 15 to 150 neural time steps (i.e., measured by the number of updates of the neural states) of starting from an initial position and ending at either end of the T-maze (or running out of the assigned time duration).

If the weights responsible for the VTEs were present among those trials, we would expect that the strength varied initially and then became stabilized toward the end of the trial as a similar observation on the VTEs. In the case of the HL robot, all the modules displayed non-periodic variations, oscillating initially to stabilize later on. The weights of IR and vision decreased progressively over all the trials. These variations show that the HL robot was changing its behavior progressively during the learning. In the L robot, no weights showed such a gradual change. The strengths of the weights looked similar both at the earlier and later stage of the learning. The L robot seemed to take the same strategy through the whole 100 trials. This analysis alone does not explain the source of the VTEs, but it implies that the VTEs were not mere oscillations of the motor neurons, but were caused by the gradual changes of their synaptic weights.

**Figure 7:**
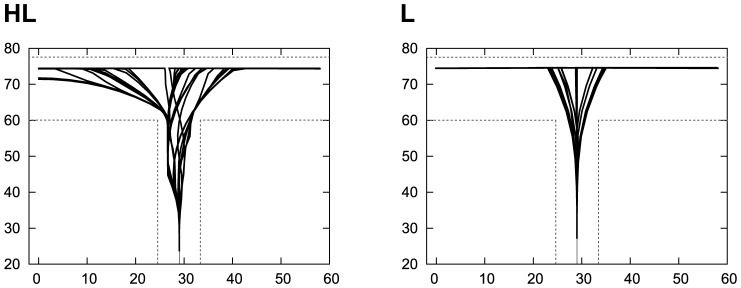
Trajectories of the robot during the 100 trials. The trajectories of 100 trials are superposed. HL: High to low VTE. L: Low VTE.

The trajectories of the robot during the 100 trials are shown in Figure 7. It can be seen that the HL robot showed unstable trajectories, while the L robot (Figure 7L) showed stable ones. This observation suggests that the orbit that the HL robot took was destabilized, while the orbit of L was stabilized. Therefore, we hypothesized that VTE is generated from chaotic activity of the neurons. To quantify the degree of chaos, we computed the maximum Lyapunov exponents (MLE), or an index of chaos, by quantifying the instability of the orbits, which is defined by the following formula;
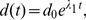
(5)where 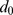 is the distance between the two neighboring points, while 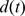 is the average divergence between the two after 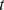 time steps. If the 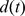 exponentially increases with time 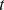, then the 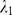 converges to a positive value, where the system is regarded as chaotic. Rosenstein et al. provided an algorithm for calculating the MLE from an experimental time series [22]. The MLE was computed by the three steps:

1. Reconstruct 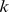 dimensional vectors 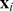 =  {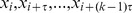} from the given time series {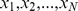}, where 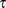 represents the time delay.
2. Choose the closest pairs of the reconstructed vectors and compute how the distance between the two develops over time. By using the fast Fourier transform (FFT), the mean period 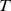 is computed, where the closest pairs are selected from vectors that are not in the same period; 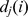 denotes the distance evolved after 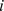
th time steps between the 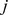
th pair of the closest pairs. The distance between two vectors (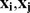) is calculated by Euclidean norm 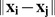.
3. The divergence rate of 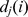 is approximated as:
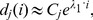
(6)where 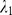 denotes the MLE. The equation above can be rewritten by:

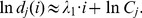
(7)The logarithm of the distance 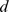 is proportional to 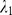, where we can estimate the value of the MLE. Therefore, 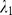 is estimated from a least-squared fit to the average line (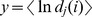), which is obtained from sampling the 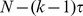 pairs.

In the current experiment, we adopted the slope of the fitted line as the MLE only when the R-squared value (i.e., the goodness of fitting) was larger than 0.8, and otherwise the MLE was set to 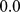. The R-squared value was computed by fitting the first 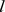 points with a line. When the estimated value was negative, then the value was set to 0.0. In general, negative Lyapunov exponents indicate the convergence speed of the two orbits, which is insignificant after reaching a stable state.

To compute MLE, we used the virtual unit 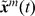 for the motor module and the differences between the virtual and state units 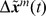 for the other sensory modules. This is because the motor value 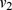 (Equation 1) was equivalent to the virtual unit of the motor 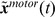 so that it was more important than the differences 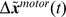 for determining the robot's behavior. For the other sensory modules, the differences 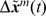 was propagated to the motor module to determine 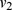 so that they were more effective on its behavior. We obtained time series of the averaged neural activity for the respective sensory or motor module and computed MLE of the time series for each 100 trials. The number of data points differed depending on trials and robots, where each time series had roughly 15–150 data points. As for parameters, we set 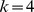, 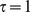, and 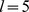.

**Figure 8:**
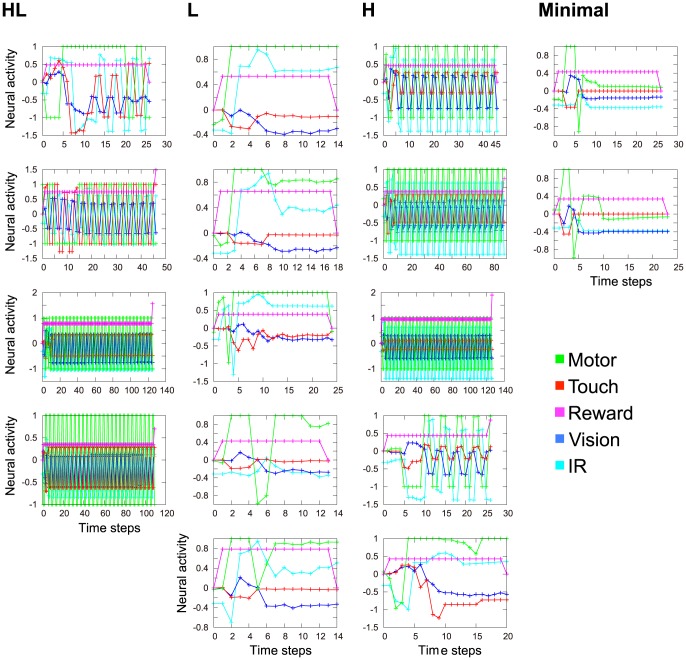
Examples of time series used for the MLE computation. The time series presented here is obtained from the 32*th* trial as an example of all the evolved 16 robots. The time series is the averaged neural activity within a same module. The virtual unit 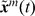 for the motor module and the differences between the virtual and state units 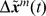 for the other sensory modules are presented. The colors of the lines represent sensory or motor modules, that is, vision: blue, IR: cyan, touch: red, reward: magenta, motor: green.

Figure 8 shows several examples of time series that were used for MLE computation. These time series were obtained from the 32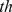 trial as examples for all the 16 evolved robots. The *x* axis shows time steps, while the *y* axis indicates the activity level of neurons. Colors represents sensory or motor modules, that is, vision: blue, IR: cyan, touch: red, reward: magenta, motor: green. This figure indicates that the neural activity appeared different depending on the type of VTE. The HL and H robots showed oscillating and unstable neural activities, while the L and Minimal robots exhibited rather stable activities.

**Figure 9:**
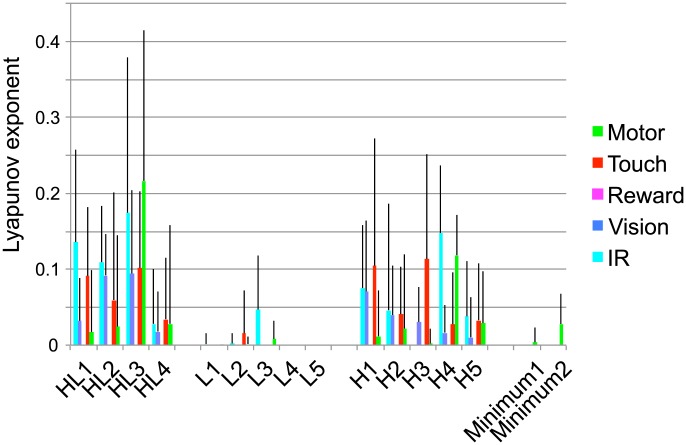
Maximum Lyapunov exponents (MLE) averaged over the 100 trials. The MLE is calculated for each five modules, and for each evolved 16 robots, which is denoted by HL1, HL2, …, Mimimal2. The time series used for estimation the MLE is an averaged neural activity for the respective modules. The error bar indicates standard deviation.

Figure 9 shows the averaged MLE over the 100 trials, where the error bar indicates standard deviation. The MLE is calculated for the respective five modules of each of the 16 evolved robots, which are denoted by HL1, HL2, …, Minimum2. We can see that HL and H robots showed positive MLEs more often than the L and the Minimal robots. This suggests that 1) Hebbian learning leads to both chaotic and non-chaotic neural activity even in the same environment and 2) chaotic activity is positively correlated with the presence of VTE, while non-chaotic activity is not.

In this paragraph, we would like to discuss the validity of the MLE estimation. We used time series that consisted of around 15–150 data points, which is quite small compared with a conventional way of MLE estimation. For instance, Rosenstein et al. estimated the MLE of Logistic map, Lorentz attractor, or Rössler attractor, by using 500–5,000 data points [22]. Enough data points are needed to see if the state converges into a chaotic attractor with a positive MLE. In this respect, our data might not have enough points to prove the convergence. However, the MLE measured the local divergence rate between neighboring pairs, so that it was still able to explain the complexity of the time series. In other words, the MLE evaluated transient chaos, which is computed from a finite number of data points. Indeed, the MLE that we obtained appeared to explain well the time series as presented in Figure 8. Additionally, to prove the plausibility of our method, we computed MLE by changing parameters (i.e., with 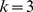 or with 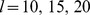), where the results exhibited the same tendency as observed above.

Note that the performance of each robot was very high (100% success) irrespective of the type of VTE. This might be seen as paradoxical because one might expect the instability to break the learned sensor and motor mappings that provide successful behavior, resulting in low performance. Therefore, we hypothesized that the robot with VTE might actively use VTE to complete the task, not just as unstable head oscillations. This is evaluated in the next paragraph by calculating the robot's performance under perturbation of VTE.

The next step of our investigation was to determine whether VTE was a mere epiphenomenon or if it had a specific role in completing the task. We took an HL robot (HL1), which is the same one as that in Figure 3HL, and artificially prevented the presence of VTE at the motor level by resetting the angular velocity to zero every time VTE was detected, forcing the robot to maintain its current direction. Our hypothesis was that if VTE is a mere epiphenomenon and not necessary for achieving the maze task, preventing it will not alter performance. On the other hand, if VTE is necessary or at least helpful for the task, the prevention of VTE will decrease performance. In order to test the hypothesis, we prevented VTE with a threshold 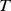. If the motor output 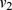 (Equation 1) changed sign and the change was larger than the threshold range 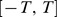, one VTE was counted. We varied the VTE threshold from 0 to 1, where threshold  = 0.0 meant that any body rotation would be regarded as VTE and they were all prevented, while threshold  = 1.0 meant that no body oscillations were prevented (the original case).

**Figure 10:**
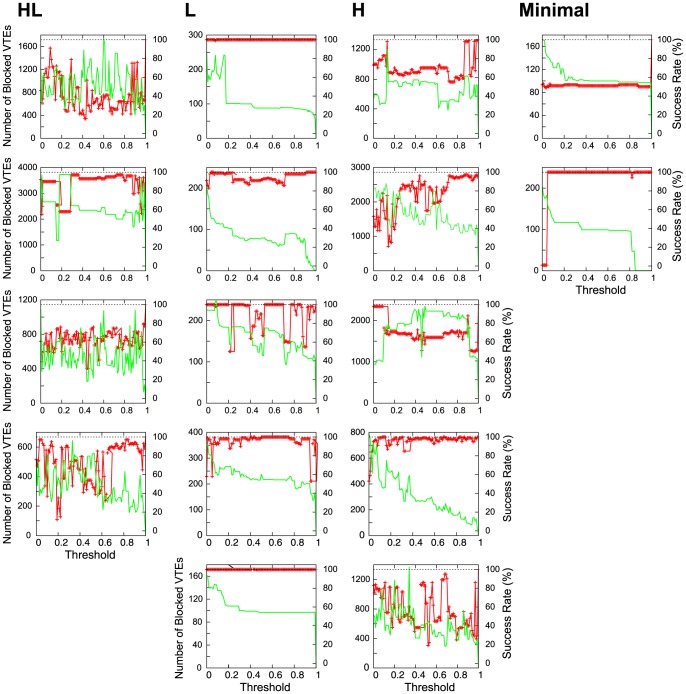
Change in performance with blocked VTE for all of the 16 evolved robots. The 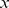 axis represents the threshold. The *y* axis with the red line shows success rate during the 100 trials, while the green line denotes the total number of blocked VTEs through the 100 trials. The black line is to guide the 100% success rates.

The results of this analysis are shown in Figure 10 for all the 16 evolved robots. Each sub-figure represents a result from each robot. The *x* axis shows the threshold value. The right *y* axis with the red line shows success rates during the 100 trials. The left *y* axis with the green line denotes the total number of blocked VTEs through the 100 trials. The horizontal black line is a guide to the 100% success rates. In all the HL robots, we found that introducing the threshold always reduced performance (Figure 10HL; red line). This result shows the absence of VTE inhibited the correct acquisition of the task while maintaining an accurate control of the robot. One might wonder why the success rate did not gradually decrease with respect to the increase of the threshold. The reason is that suppression of VTE caused nonlinear effects on the robot's behavior. Once a VTE was suppressed, the robot received different sensory signals from the original setups, where the resulting behavior did not monotonically decrease.

Additionally, we found that VTE did not always affect learning performances. The second and third columns of Figure 10 show the results of the L and H robots, respectively, where some of them did not change their performances even with the suppression of VTE (red line in Figure 10). The green line in Figure 10 shows that a certain amount of VTEs were suppressed in those robots. This result suggests that, for those robots, VTE was acting as mere head oscillations that were not necessary in achieving the task. In contrast, for the HL robots, VTE helped them to achieve higher performances. From these observations, we say that VTE is the result of L (and some H) robots' learning behavior but it is the cause of HL robots learning a cue-reward relationship.

We considered that VTE observed in the HL robots was used actively to complete the T-maze task. This result is consistent with experiments of real rats and humans, where better performance is found in the presence of VTE [2], [6]. In the next section of this paper, the usefulness of VTE is linked to the robustness of the behavior.

### Robustness of VTE

To investigate the robustness of the robot's behavior for each types of VTE, we analyzed its performance under varying initial conditions. During evolution, the starting position of the center of the body was fixed to 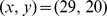, as shown in Figure 1. This experiment explored whether perturbations to the starting position affected the performance by testing the robot from different starting positions inside the central arm of the T-maze. The robot repeated the task 100 times from different initial positions by changing (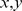) as 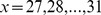 and 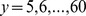 (280 initial positions in total). We calculated the success rates for each initial position.

**Figure 11:**
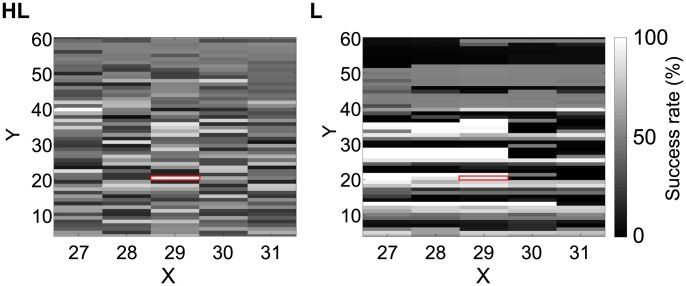
Examples of the average success rates for each starting positions. Each grid cell is filled with a colour representing the success rates when a robot starts the task from within the grid cell. The original starting position used for the GA was 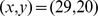, which is marked by red lines. From this position, the robot obtained 100% success rate. HL: High to low VTE. L: Low VTE.

**Figure 12:**
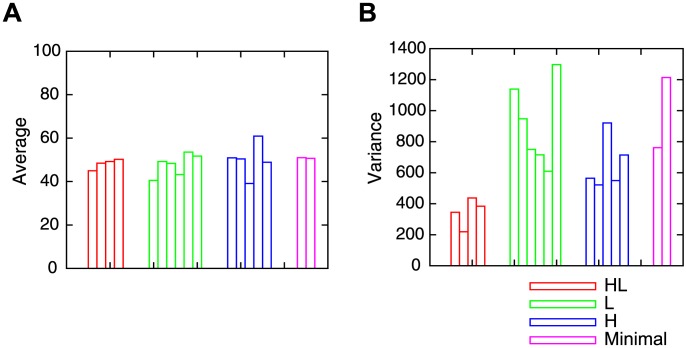
Average and variance success rates over all the initial positions 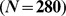. Red, green, blue and pink bars indicate the results of HL, L, H and Minimal respectively. A: Average success rate. B: Variance of success rate.

Figure 11 shows the results of the HL and L, respectively. The *x* and *y* axis indicate the coordinates of the central arm of the T-maze. Each pixel of the figure indicates the success rate when the robot started the 100 trials from this position. The original starting position used for the GA was 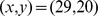, which is marked by red lines in this figure. From this position, the robot obtained a 100% success rate. This figure shows that the performance was not constant for all the initial positions and allows a comparison of the variation in performance between the two models. Figure 11HL gives an example of HL, where the robot mainly obtained around 50% success rates, with several initial positions leading to success rates of 100% or below 20%. On the other hand, Figure 11L shows an example of L, where the robot obtained mainly a success rates of 100% or 0% with several initial positions giving around a 50% success rate, suggesting a higher variance of the success rate. This tendency is summarized by Figure 12, which shows the average and the variance of the success rates for every evolved robot. Figure 12A indicates the average of the success rates, showing that the types of VTE do not have varying impacts on the average success rates. On the other hand, Figure 12B shows the variance of the success rates – the HL robots kept the variance under 400 (red line), while the other three groups had a variance above 550 (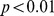; student *t*-test). Therefore, despite the fact that four groups had a similar average performance, the HL robots withstood changes in its initial position, while the other types were strongly affected by those changes. This result suggests that the presence of the HL type of VTE, which is similar to that of the rats [1], [4], is associated with a higher level of robustness. Animal experiments could be performed to test this hypothesis. Robustness acquired by VTE also explains why the prevention of VTE lowered the overall performance of the robots. Namely, preventing VTE will reduce robust control and destabilize the behavior.

**Figure 13:**
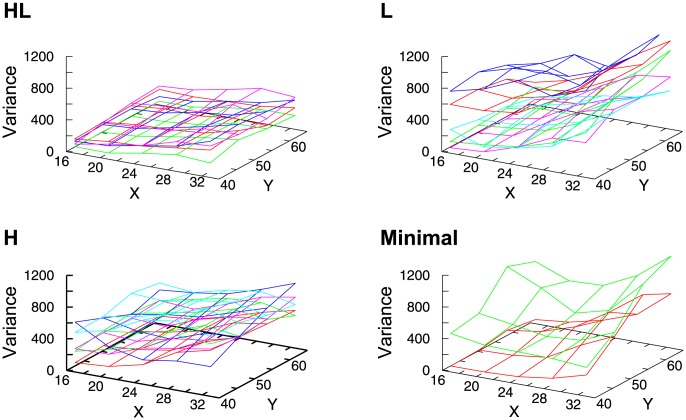
Examples of the variance of the success rates with different environmental size. The 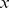 and *y* axis show the size of X and Y in the figure 1. The 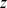 axis shows the variance. HL: High to low VTE. L: Low VTE. H: High VTE. Minimal: Minimal model.

To further investigate the robustness of the evolved controllers against environmental change, we carried out the same experiments with different T-maze sizes. We varied the length of the width and height of the T-maze and calculated the average and the variance of the success rates for every starting position. Figure 13 shows the variance of the success rate, where the HL robots had a low variance of performance, while the robots in the other three groups were affected by a slight change in environmental size. This result confirms that the presence of VTE can be an indicator of the robustness of behavior.

### Effective Use of Hebbian Learning

We have shown that VTE is used actively to achieve the T-maze task, rather than serving as mere oscillations of the head direction. This leads to the question of why the destabilized orbit by VTE guarantees the best performance of the robot. We hypothesized that the destabilization by VTE enables continuous learning of the environment, while too much stable dynamics found in non-VTE robots creates a loss of adaptability in learning. To investigate this hypothesis, we conducted the following two experiments:

1) First, we varied the position of the reward by moving it further away within the same branch of the T-maze. This means that the robot could not get the reward in the expected position. If the robot was continuously gathering information from the environment, then this setup would create a contradiction between the expectation and the sensory information. On the other hand, if the robot was solving the maze just by reflex to the tactile cue, then it would still be successful, regardless of the reward position.

**Figure 14:**
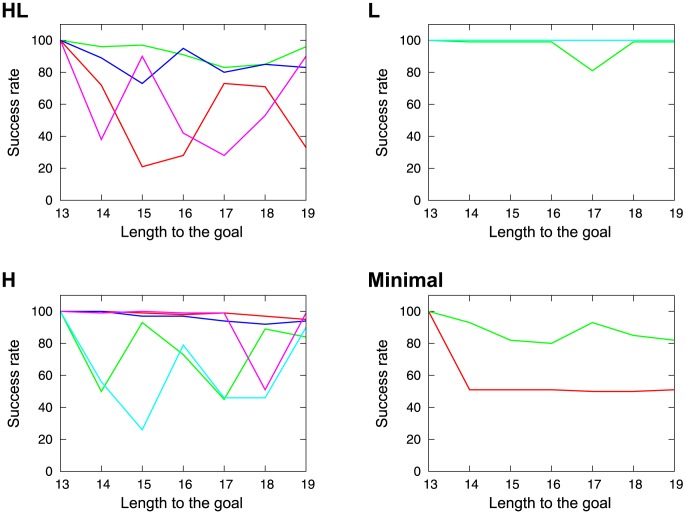
Success rates with longer distance from the tactile cue to the reward. The 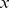 axis shows the length to the goal, while the *y* axis indicates the success rates. HL: High to low VTE. L: Low VTE. H: High VTE. Minimal: Minimal model.

The results of this experiment are shown in Figure 14: all the HL robots and some H robots reduced their success rates (Figure 14HL and H), while most of the L and Minimal robots maintained their high success rates (Figure 14L and Minimal). As these results suggest, the HL robots modified their behavior by continuously gathering information from their environment. On the other hand, the behavior of the non-VTE robots (L and Minimal) was only dependent on the cue command, and not on the reward position, so that they seemed to just obey the cue command, which is reflective rather than ongoing decision making.

2) Second, to evaluate the effect of learning, we compared the performance of the robots with and without learning. Originally, the synaptic weights were continuously updated through the 100 trials so that the robot started each set of the 100 trials with different initial weights. Therefore, we had 100 initial weight sets, one for each corresponding trial. In the no-learning condition, the robot started each 100 trials with an initial weight set corresponding to the trial, where the synaptic weights were fixed and not updated. In the learning condition, a robot updated its weights in the same way as the original experiment. For each trial, we computed the performance by perturbing the initial conditions, similar to what we did previously for computing the robustness; that is, the initial position was changed for every grid inside the central arm of the T-maze.

Sixteen examples of these results are shown in Figure 4. In this figure, the results from the respective 16 robots are presented. The *x* axis denotes trials and has auxiliary lines every 10 trials. The scale of each axis is set to the same value in every figure. The red line shows the success rates with learning, while the green line indicates success without learning. The black line indicates the number of VTEs observed in the original condition (i.e., with learning activated and from the original starting point; the same condition as Figure 3). For the HL and H robots, the performance was worsened or almost the same when the learning was deactivated. On the other hand, the L and Minimal robots improved performance without learning. This tendency suggests that the robots with VTE utilized learning to maintain their performance, while, for those without VTE, the learning process did not work properly.

We have therefore concluded that the destabilized orbit generated from VTE allows continuous learning by gathering information from the environment, which means that the robots are performing embodied cognition, that is, ongoing generation of their behavior from the interaction between the body and the environment. On the other hand, the robots without VTE are not embodied in their environment, which does not allow ongoing learning.

## Discussion

VTE is a behavior observed in experiments with rats that seems to suggest self-conflict [1], [23], [24]. It is mainly observed when rats are uncertain about making a decision, for example, when they make a mistake or change their strategy [5]. The presence of VTE is regarded as an indicator of a deliberative decision-making process, that is, the process of searching, predicting and evaluating future outcomes [4]. Deliberative decision making is an opposite notion to an automated decision-making process, such as habituation or reflex, and is computationally slower, while it can allow ongoing control to achieve flexible behavior. In fact, better performance is found when animals show VTE [2], [6].

In this paper, we have tried to show the underlying mechanism responsible for the observation of VTE and the advantages provided by it to demonstrate that VTE is associated with the chaotic activity of neuronal dynamics (Figure 7 and 9). We modeled VTE in a simulated robotic experiment based on [16], which is a simplified abstract model of learning beings. Our aim is not to imitate the biological structure of animals, but to construct an abstract model to understand the essential mechanism that causes VTE. We expected that this simplified model would show 1) an emergent property of VTE just from a body-environment interaction, 2) the necessary mechanism behind VTE, and 3) the effects of VTE on behavior.

As we have seen in this paper, the spontaneous learning of the correlation between a cue and the location of the goal is achieved after repeatedly exploring the environment. We found that some robots showed conflicting behaviors similar to biological VTE patterns reported in experiments done with rats [1], [4]. A common feature is that the highest frequency of VTE was found at the beginning of the learning stage and gradually diminished after mastering the task (Figure 3). Especially at the sixth or seventh trial, which were just after switching the reward position, the robot showed the highest number of VTEs. This could be the results of the interaction between the two neuronal pathways mentioned earlier in the method section; that is, one path (reward – vision – motor) leads the robot to go to the previous reward position (wrong decision), and the other (tactile – motor) lets it follow the tactile cue (correct decision). Therefore, we concluded that the head movements observed here were similar to real VTE, because they appear to be elicited from a conflict, rather than mere oscillations caused by immature synapses. In the later analyses, we collected more evidences that the VTE found in this experiment was not just an epiphenomenon expressed through head oscillations, but actually improved the learning performance of the robot.

We found that with different parameters the robot showed other patterns of VTE, which we classified into three groups: high at the beginning and low afterward (HL), low during the entire learning period (L), and high all the time (H). Those robots belonging to the L and H groups could have different strategies from the one explained in the method section. Interestingly, VTE was found only in some neural networks with redundant connectivity, while networks with a minimal ensemble of connections failed to show VTE.

**Figure 15:**
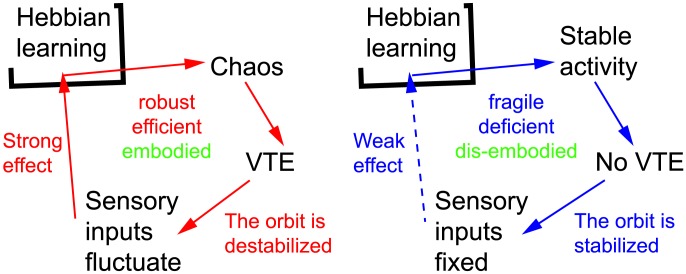
Diagrams showing overall dynamics with and without VTE. The path with VTE (left; red line) exhibits the following. 1) Neurons showed chaotic activity, which destabilises the orbit, making sensory inputs fluctuate and vary constantly. 2) When the chaotic dynamics is maintained, it prevents the robot from falling into a stable attractor. 3) The robot repeatedly learns the same environment under different conditions by exploring different paths, allowing flexible and embodied control of behavior, resulting in robust efficiency. A robot not showing VTE (right; blue line) displays a stable activity of its neurons, leading to a periodic sensory input pattern. This fixed pattern of sensory inputs gives only a weak effect on Hebbian leaning, which results in a fragile reaction to environmental perturbations.

Figure 15 summarizes the two learning processes, with and without VTE. A learning process with VTE (Figure 15 left; red line) will go through several steps. First, neurons display chaotic activity, which creates an exploratory motion pattern that allows the sensory inputs to fluctuate and vary constantly. When the chaotic dynamics is maintained, it prevents the robot from falling into a stable attractor. Therefore, the robot repeatedly learns the same environment under different conditions by exploring different paths. This prevents the convergence of the synaptic weights, allowing adaptive control of the behavior (i.e., if an unpredictable event happens, the robot can smoothly adapt to it; Figure 14 and 4). We believe that these are the advantages of the VTE, as can be seen in Figure 11, 12, and 13. On the other hand, a robot not showing VTE (Figure 15 right; blue line) displays stable neuron activity, which lacks the chaotic instability, leading finally to settling into a periodic motion behavior. Such a stable behavior is not conductive to adaptability in general. Therefore, we have verified that the presence of VTE endows the robot with the ongoing control necessary to achieve adaptive behavior. Different from a mere reactive behavior, a robot showing VTE can adjust its behavior to the task, thereby improving its behavioral pattern. The ongoing learning found in the VTE-showing robots is also a feature of deliberative decision making. Therefore, it is plausible to say that our experiments computationally supports the idea that VTE is a behavioral representation of the deliberative decision making process [4], [5].

We can paraphrase the above observation by making the following statement: VTE adds “internal” noise generated by chaotic activity in the neural network to the system's behavior so that the performance is automatically geared toward robustness. As a consequence, robots without VTE fail to extrapolate the correct behavior from inexperienced sensory inputs. VTE is associated with the chaotic activity of neurons, where Hebbian learning continuously reorganizes the connectivity patterns, which results in robust behaviors. Namely, chaotic activity in the neural network causes sensory fluctuations, which results in robust and adaptive behavior, where VTE is a behavioral representation of the internal fluctuations.

As mentioned in the introduction, VTE is potentially regarded as a behavioral representation of deliberative decision making, which is the opposite notion of reactive decision making [25], [26]. Here, we discuss connections of our study with the previous studies on the deliberation process. We propose in this paper that chaotic activity in the neural network produces behavioral VTE which causes sensory fluctuations that are crucial for learning and adaptation. In this respect, VTE is an emergent property of the system that is not necessarily deliberately triggered by the agent. Still, although indirectly, VTE in this paper shares several important features of deliberative decision making, that is, 1) allowing ongoing control of behavior, which is differentiated from a mere reflection, and 2) it is observed in uncertain conditions. Those two features are summarized as follows:

1. **Allowing ongoing control**: As presented in the result section (Figure 14), the HL robots changed their behavior if the reward position differed from the original one, suggesting that the robots modified their behavior by gathering information on an ongoing basis through the learning process. On the other hand, the behavior of the L robots was only dependent on the cue command, not on the reward position, which suggests that they were just reflective to the cue. Additionally, Figure 4 showed that the L robots improved their performance when the learning was off, while the HL robots worsened / maintained its performance. This also supports the idea that the HL robots learned behavior on an ongoing basis, while the L robot did not learn properly. From these observations, we would like to say that ongoing learning, differentiated from reactive decisions, can be self-organized through low-level body-environment and sensory-motor interactions. Although it is difficult to say our robots acted based on deliberative decision making, our results at least suggested that the VTE-showing robots acted in a more complex way than merely displaying reactive behavior.
2. **Observed in uncertain conditions**: As described earlier in our discussion, VTEs in the HL robots were observed just after the first switch of the reward position (the sixth and seventh trials). After the first switch, the robots were expected to face the two conflicting strategies: the reward guided to the previous reward position and the tactile cue indicating the current reward position. Therefore, it is plausible to say that the HL robots displayed VTE in the most uncertain conditions. This feature – VTE is observed in uncertain conditions – is shared by the VTE observed in rodent experiments [5]. We put the robot in different experimental arenas (i.e., different size of arena; Figure 13), and found the HL robots achieved robust control, which suggests that the VTE provided adaptive control under uncertain conditions. The adaptive control observed in the HL robots may be explained by Figure 7, which indicates that the HL robots visited a broader area than the L robots. The greater exploration of the arena by the HL robots could grant them with higher robust control.

In those two respects, our VTE model shares several important features of deliberative decision making observed in rodent experiments. Namely, the VTE observed in this paper can be a behavioral representation of deliberative decision making, as well as being an emergent property. We therefore have demonstrated that low-level body-environment and sensory-motor interactions can generate conflicting options in the central nervous system (as suggested in the methodology section and Text S1 and Figure S1–S4 in File S1), resulting in VTE finally switching into non-VTE phases. In other words, higher-level model-based navigation can emerge through lower-level interactions.

Our results support the idea that VTE can be a behavioral representation of deliberative decision making from the view of a computational approach. Here, we discuss the validity of our way of modeling VTE by comparing previous computational models. There are several previous computational models designed to explain the switch from deliberative to reactive behavior [27]–[29]. Although these models do not explicitly model VTE, their results demonstrated that behavioral sensitivity, such as VTE, could be observed in uncertain conditions. As an important difference from our model, their models used specific criteria, for example, an uncertainty level on action values, to induce the switch. On the other hand, our robots gradually reduced the amount of VTE, where the switch (i.e., higher VTE at the beginning and less afterward) generated spontaneously from lower-level interactions between the sensors, body, and environment, that is, from embodied properties. Thus, our results suggest that embodiment allows adaptive and intelligent behavior, like deliberative decision making, without any designed parameters in advance.

In this respect, our model can be differentiated from conventional methods for controlling robots (e.g., [29], [30]). The parameters of the robot were evolved through GA, and based on the parameters, the robot had to learn the task by Hebbian learning. Hebbian learning is focused on the autonomous and on-line control of the robot's movements, which we believe to be a biologically plausible learning mechanism. Contrary to a widely used method for controlling robots (e.g., back propagation, which tunes synaptic weights to minimize the error between the expected outputs and its own [29], [30]), Hebbian learning does not require any explicit functions. Due to the lack of an evaluation function, it is difficult to control robots to solve complex tasks, yet Hebbian learning is an important piece of the mechanisms responsible for the capacity to learn and to memorize information. We aimed at creating a simple and abstract model of VTE so that this model does not necessarily have counterparts observed in human or rodent brain systems [31]–[34]. Also, the network structure we used here was specific to the Bovet-Pfeifer model. Still, we think the obtained results do not depend on the detailed architecture of the model. However, we believe that the separation of Hebbian learning from the internal dynamics is an important feature of the model, which is necessary in order to reproduce these results.

To conclude, we believe that linking explorative motions like VTE, chaotic activity, and robust learning is the way to understand how Hebbian learning functions in the real brain system. The connection between robust learning and VTE is consistent with previous research [2], [6], in which rats/humans were found to exhibit a better performance when they showed VTE. Perhaps the biologically missing piece is whether chaos is truly part of the mechanisms involved in how the real brain works [35]. We believe that VTE is an essential phenomenon for understanding how the brain works, but as it is still important to determine whether chaos is an epiphenomenon caused by VTE or the mechanism that causes VTE, the relationship between chaos and VTE must be carefully investigated further in animal experiments (e.g. [3]–[5]).

## Supporting Information

File S1 **Text S1 and Figures S1–S4.**
(PDF)

## References

[1] Tolman EC (1939) Prediction of vicarious trial and error by means of the schematic sowbug. Psychol Rev 46: 318–336.

[2] Hu D, Amsel A (1995) A simple test of the vicarious trial-and-error hypothesis of hippocampal function. Proceedings of the National Academy of Sciences of the United States of America 92: 5506–9. 7777539 10.1073/pnas.92.12.5506 PMC41724

[3] Johnson A, Redish AD (2007) Neural ensembles in ca3 transiently encode paths forward of the animal at a decision point. The Journal of neuroscience: the official journal of the Society for Neuroscience 27: 12176–89. 17989284 10.1523/JNEUROSCI.3761-07.2007 PMC6673267

[4] van der Meer MA, Johnson A, Schmitzer-Torbert NC, Redish AD (2010) Triple dissociation of information processing in dorsal striatum, ventral striatum, and hippocampus on a learned spatial decision task. Neuron 67: 25–32. 20624589 10.1016/j.neuron.2010.06.023 PMC4020415

[5] Schmidt B, Papale A, Redish AD, Markus EJ (2013) Conflict between place and response navigation strategies: Effects on vicarious trial and error (vte) behaviors. Learning & Memory 20: 130–138. 23418392 10.1101/lm.028753.112

[6] Voss JL, Warren DE, Gonsalves BD, Federmeier KD, Tranel D, et al (2011) Spontaneous revisitation during visual exploration as a link among strategic behavior, learning, and the hippocampus. Proceedings of National Academy of Sciences 108: E402–E409. 10.1073/pnas.1100225108 PMC3150890 21768385

[7] Tarsitano M (2006) Route selection by a jumping spider (portia labiata) during the locomotory phase of a detour. Animal Behaviour 72: 1437–1442. 10.1006/anbe.1999.1138 10458876

[8] Rossler OE (1994) Fraiberg–lenneberg speech. Chaos, Solitons & Fractals 4: 125–131.

[9] Ikegami T (2007) Simulating active perception and mental imagery with embodied chaotic itinerancy. Journal of Consciousness Studies 14: 111–125.

[10] Johnson A, Varberg Z, Benhardus J, Maahs A, Schrater P (2012) The hippocampus and exploration: dynamically evolving behavior and neural representations. Frontiers in human neuroscience 6: 216. 22848196 10.3389/fnhum.2012.00216 PMC3404547

[11] Harvey I, Husbands P, Cliff D, Thompson A, Jakobi N (1997) Evolutionary robotics: the sussex approach. Robotics and Autonomous Systems 20: 205–224.

[12] Nolfi S, Floreano D (2000) Evolutionary Robotics: The Biology, Intelligence, and Technology of Self-Organizing Machines. MIT press.

[13] Brooks RA (1991) Intelligence without representation. Artificial Intelligence: Building Embodied, Situated Agents 47: 139–159.

[14] Pfeifer R, Scheier C (1999) Understanding intelligence. MIT Press.

[15] Pfeifer R, Bongard JC (2006) How the body shapes the way we think. MIT press.

[16] Bovet S, Pfeifer R (2005) Emergence of delayed reward learning from sensorimotor coordination. In: IEEE/RSJ International Conference on Intelligent Robots and Systems(IROS). Edmonton, pp. 841–846.

[17] Iizuka H, di Paolo E (2008) Extended homeostatic adaptation: Improving the link between internal and behavioural stability. In: Proceedings of the 10th International Conference on Simulation of Adaptive Behavior. Berlin, Germany: Springer-Verlag, pp. 1–11.

[18] Mondada F, Bonani M, Raemy X, Pugh J, Cianci C, et al.. (2009) The e-puck, a robot designed for education in engineering. In: Proceedings of the 9th Conference on Autonomous Robot Systems and Competitions. pp. 59–65.

[19] Bovet S (2006) Emergence of insect navigation strategies from homogeneous sensorimotor coupling. In: Proceedings of the 9th International Conference on Intelligent Autonomous Systems. pp. 525–533.

[20] Holland JH (1975) Adaptation in Natural and Artificial Systems. Ann Arbor: The University of Michigan Press.

[21] Bovet SI (2007) Robots with Self-Developing Brains. Ph.D. thesis, Universität Zürich.

[22] Rosenstein MT, Collins JJ, Luca CJD (1993) A practical method for calculating largest lyapunov exponents from small data sets. Physica D 65: 117–134.

[23] Muenzinger KF, Gentry E (1931) Tone discrimination in white rats. J Comp Psychol 12: 195–206.

[24] Muenzinger KF (1938) Vicarious trial and error at a point of choice: A general survey of its relation to learning efficiency. Journal of Genetic Psychology 53: 75–86.

[25] Khamassi M, Humphries MD (2012) Integrating cortico-limbic-basal ganglia architectures for learning model-based and model-free navigation strategies. Frontiers in Behavioral Neuroscience 6. 10.3389/fnbeh.2012.00079 PMC3506961 23205006

[26] van der Meer M, Kurth-Nelson Z, Redish AD (2012) Information processing in decision-making systems. Neuroscientist 18: 342–359. 22492194 10.1177/1073858411435128 PMC4428660

[27] Daw ND, Niv Y, Dayan P (2005) Uncertainty-based competition between prefrontal and dorsolateral striatal systems for behavioral control. Nature Neuroscience 8: 1704–1711. 16286932 10.1038/nn1560

[28] Keramati M, Dezfouli A, Piray P (2011) Speed/accuracy trade-off between the habitual and the goal-directed processes. PLoS Computational Biology 7: e1002055. 21637741 10.1371/journal.pcbi.1002055 PMC3102758

[29] Caluwaerts K, Staffa M, N'Guyen S, Grand C, Dollé L, et al (2012) A biologically inspired metacontrol navigation system for the psikharpax rat robot. Bioinspiration & Biomimetics 7 025009: 1–52. 10.1088/1748-3182/7/2/025009 22617382

[30] Rumelhart DE, McClelland JL (1986) Parallel distributed processing: explorations in the microstructure of cognition. MIT Press. 10.1111/cogs.12148 25087578

[31] Daw ND, O'Doherty JP, Dayan P, Seymour B, Dolan RJ (2006) Cortical substrates for exploratory decisions in humans. Nature 441: 876–879. 16778890 10.1038/nature04766 PMC2635947

[32] Cohen JD, McClure SM, Yu AJ (2007) Should I stay or should I go? How the human brain manages the trade-off between exploitation and exploration. Philosophical Transactions 362: 933–942. 10.1098/rstb.2007.2098 PMC2430007 17395573

[33] Barnes TD, Kubota Y, Hu D, Jin DZ, Graybiel AM (2005) Activity of striatal neurons reflects dynamic encoding and recoding of procedural memories. Nature 437: 1158–1161. 16237445 10.1038/nature04053

[34] Humphries MD, Khamassi M, Gurney K (2012) Dopaminergic control of the exploration-exploitation trade-off via the basal ganglia. Frontiers in Neuroscience 6: 1–14. 22347155 10.3389/fnins.2012.00009 PMC3272648

[35] Tsuda I (2001) Toward an interpretation of dynamic neural activity in terms of chaotic dynamical systems. Behavioral and Brain Sciences 24: 793–847. 12239890 10.1017/s0140525x01000097

