# Supplementary material for: A Robotic Approach to Understanding the Role and the Mechanism of Vicarious Trial-And-Error in a T-Maze Task
Source: PLoS One. 2014 Jul 22;9(7):e102708. doi: 10.1371/journal.pone.0102708 (PMC4106851; doi:10.1371/journal.pone.0102708)
Supplement: File S1 — Text S1 and Figures S1–S4. (PDF) [file pone.0102708.s001.pdf]

Supporting Information (Text S1) – Development of the neural network

A reward-oriented behavior emerges through Hebbian learning. In this section, we explain the underlying mechanism of the emerged behavior. As described in the method section, we found several possible parameter sets by the genetic algorithm to maximise the fitness value. Although those different parameter sets might result in various strategies, here we describe the most possible mechanism to generate the reward seeking behavior of the robot, by following Bovet's description [16, 21]. The way of the description is almost same as what is written in his Ph.D. thesis [21], but we think it might be difficult to access it. Therefore, we describe the mechanism here in this supporting information.

As is noted in the method section, the experiments took place in two phases: a training phase and a testing phase. In the training phase, the robot learned a simple correlation between the IR, vision and motor modules. In the testing phase, the robot was required to solve a reward seeking task in a T-shaped maze. Here we describe first the learning mechanism of the training phase, and afterward the testing phase.

Training phase

During the training phase, the robot explored the maze with the cue and reward deactivated and with the motor speed fixed to the two variables $v_2 = (1, 0)$ or $(0, 1)$, which induce left and right turns respectively. When it touched a wall, the robot was returned to its initial position and the motor speed was switched to the other value. This process was repeated 20 times. The purpose of this phase was to give the robot a chance to learn the correlations between the IR, vision, and motor modules. Especially, the "IR – motor" and "vision – motor" path are important to solve the succeeding test phase; The "IR – motor" path is able to induce wall avoidance behaviour, and the "vision – motor" path gives the robot an important association between optical flow and turning speed. In this paper, optical flow means the difference between the current visual image and the image τ time units previously. The detailed descriptions are in the next parts.

"IR – motor" path

This path lets the robot avoid the walls. As is noted in the method section, the neuron has two components, i.e., the virtual and the state units. Activity patterns for each virtual and state units are illustrated in Fig. S1. In this figure, each module has five boxes, which corresponds to the five types of variables important for computing the neural network in Eq. 2 and 3 in the original manuscript, i.e.,

1) the state unit at the current time step $\boldsymbol{x}^m(t)$,

2) the state unit τ step before $\boldsymbol{x}^m(t-\tau)$,

3) the difference of those two $\Delta\boldsymbol{x}^m(t) = \boldsymbol{x}^m(t) - \boldsymbol{x}^m(t-\tau)$,

4) the virtual unit at the current time step $\tilde{\boldsymbol{x}}^m(t)$,

5) the difference between the virtual and state units $\Delta\tilde{\boldsymbol{x}}^m(t) = \tilde{\boldsymbol{x}}^m(t) - \boldsymbol{x}^m(t)$.

The IR module has circles in each of the five boxes, which denote activity levels of each of the six IR neurons. The left three circles corresponds to the left side of the IR sensors, while the right ones are for the right IR sensors. As for the motor module, the arrows inside the five boxes indicate the direction of turning. The virtual unit of the motor module $\tilde{\boldsymbol{x}}^{motor}(t)$ is used for calculation of the actual turning speed (Eq. 1).

When the robot turns toward a wall (Fig. S1A), to the left wall for example, this causes a correlation between an increase of the left part of the IR proximity signals and turning left. Therefore the robot learns a positive correlation between $\Delta \boldsymbol{x}^{IR}(t)$ with the left part positive and motor activity $\boldsymbol{x}^{motor}(t)$ corresponding to turning to left (Eq. 2). Conversely, turning away from the left wall (Fig. S1B) causes a correlation between a decrease of IR signal in the left sensors and turning to right. Therefore, the robot will learn a positive correlation between turning left and the left part of the IR signal, and a negative one between turning right and the left part of the IR signal.

After learning this correlation, wall avoidance behavior is induced by the following process (Fig. S1C):

1. The robot makes a turn to the left wall, and gets close to it.
2. The left part of the IR signals increases.
3. Elements of the state unit $\boldsymbol{x}^{IR}(t)$ corresponding to the left IR sensors take positive values.
4. The virtual unit $\tilde{\boldsymbol{x}}^{IR}(t)$ is normally not activated, as there will be no learned correlation from the other modules to the IR module.
5. The difference between the virtual and state unit $\Delta\tilde{\boldsymbol{x}}^{IR}(t)$ $(= \tilde{\boldsymbol{x}}^{IR}(t) - \boldsymbol{x}^{IR}(t))$ has therefore its elements negative, which propagates through the positive connection from the IR module to the motor module learned above, and cause a turn to the opposite direction, i.e. the right turn.

Therefore, when the robot comes close to the wall, this "IR – motor" path causes a turn in the opposite direction, so that it can result in wall avoidance behavior.

"Vision – motor" path

This path lets the robot learn a correlation between optical flow and turning speed. In this paper, optical flow means the difference between the current visual image and the image τ time units previously (τ is the time delay in Eq. 2 and 3). The omni directional camera receives a grayscale image of the environment, so that it detects the black wall at the back and the white walls in the other sides of the T-maze. We also use the phrase "optical flow" to refer to the movement of the black wall image. For instance, if the image of the black wall moves in the right direction in its visual field, then we call it optical flow in the right direction. By this "vision – motor" path, the robot can learn a correlation between optical flow and turning speed, i.e., turning left is correlated with optical flow in the right direction, and vice versa. This correlation is used in the testing phase.

Testing phase

After learning the basic correlation between the IR, vision and motor modules, the robot starts the testing phase, i.e., solving the T-maze. The reward is placed in the left or right arm of the T-maze and changes its position every five trials, but for simplicity, we only describe here the case with the reward on the left arm. As described in the method section, two paths are important for generating the reward seeking behavior. One is the "reward – vision – motor" path, and the other is the "tactile – motor" path. Those two paths can conflict with each other after the reward position changes, which will cause VTE behavior. In the following, we describe the detailed neural activity.

"Reward – vision – motor" path

This path leads the robot to go to the previous reward position. Therefore, if the reward position is fixed, the robot can get the reward. But it is not sufficient to reach the reward when the reward position switches. Fig. S2 illustrates the processes of two example trials. At the beginning of the trials (Fig. S2A), the robot is placed on the starting point at the center arm of the maze, where the omni directional

camera detects the black wall at the back. Then, the robot goes straight, because of the constant forward velocity (Eq. 1). The whisker sensors start detecting the tactile cue on the left side (Fig. S2B). Hebbian learning captures a correlation between the increase of the tactile stimulation on the left side and sensory signals of the other modules (i.e., $\Delta\boldsymbol{x}^{tactile}(t)$ and $\boldsymbol{x}^{m}(t)$ in Eq. 2; m represents modules other than the tactile one). After a short time, the robot reaches the intersection point, and the whiskers break contact with the tactile cue. There, the whiskers detect the decrease of the tactile signal, which cancels out most of the previously learned correlations. At the first trial, the robot does not learn much correlations about the tactile cue and it makes no large turn, so that the robot once hits the back wall. But a slight difference of the right and left motors, because of asymmetry from the training phase, leads the robot to one side of the T-maze. Therefore, it reaches the reward or punishment only by chance. We explain the case of the reward here, but the reverse situation, where the robot gets to the punishment side, happens in an analogous way.

Fig. S3A shows neural activity of the reward, vision and motor modules when the robot gets the reward at the first trial. The notation is the same as in Fig. S1. The five boxes inside the vision module denote activity patterns of its visual field for the state and virtual units (upper three boxes), and the optical flow for the state difference $\Delta\boldsymbol{x}^{vision}(t)$ and the virtual difference $\Delta\tilde{\boldsymbol{x}}^{vision}(t)$ (lower two boxes). As for the reward module, the sign inside the boxes indicates the activity level. As described in the method section, the virtual unit of the reward module $\tilde{\boldsymbol{x}}^{reward}(t)$ is always set to 1.0, which is illustrated by the black triangle.

If it reaches the reward, the robot gets an increase of the reward signal, and at the same time the camera detects the black wall on the right side of its visual field. There, the robot learns a positive correlation between $\Delta\boldsymbol{x}^{reward}(t)$ and $\boldsymbol{x}^{vision}(t)$ by Eq. 2 (Fig. S2C and S3A). This correlation stores the momentary camera image when the robot gets the reward, and the image gets remembered by the reward virtual unit $\tilde{\boldsymbol{x}}^{reward}(t)$, which is described next.

After getting the reward, the robot is returned to the starting position to start the second trial (Fig. S2D and S3B). By Eq. 3, the signal from the reward virtual unit first propagates through the synaptic connectivity to the vision module, as described in the previous paragraphs. Because the synaptic weights are positive, the signal from the reward virtual unit induces a similar activity pattern of the camera image at the reward point in the first trial, i.e., the black wall image on the right part of its visual field. Therefore, the virtual unit $\tilde{\boldsymbol{x}}^{vision}(t)$ has black on the right visual field. At the same time, the actual camera image, or the state unit $\boldsymbol{x}^{vision}(t)$, has its central visual field black, because the robot is facing the black wall. Therefore, the difference $\Delta\tilde{\boldsymbol{x}}^{vision}(t)$ (i.e., the difference between the virtual and state units) has a visual flow in the right direction. By Eq. 3, this optical flow in the virtual unit propagates into the motor module to induce a left turn, because of the correlation from the right optical flow to the left turn, which is learned in the training phase (Fig. S2D and S3B). Therefore, at the second trial, the robot is again successful. If the robot goes to the punishment at the first trial, a similar process makes the robot reach the reward side at the second trial.

So far, it becomes obvious that, by using the reward, vision and motor modules, the robot goes to the same side as the reward side during the first trial. But the robot cannot succeed when the position of the reward switches. To overcome this, the robot has to learn the correlation between the tactile cue and the reward. Next, we describe the learning with the whisker sensors.

"Tactile – motor" path

This path lets the robot follow the tactile cue which indicates the correct reward position. Here we explain the second trial, i.e., the trial after the robot learns the "reward – vision – motor" path, which leads it to turn to the previous reward position (Fig. S2D). Fig. S4A illustrates the learning process of this correlation. The sign in the boxes at the tactile module denotes reward(+), punishment(-) and nothing(0) for the left and right sides of the whiskers. In Fig. S4A, the robot makes a left turn because of the "reward – vision – motor" path described above and, at the same time, it breaks contact with the

tactile cue. Therefore, by Eq. 2, the robot learns a correlation between the decrease of the state unit $\Delta\boldsymbol{x}^{tactile}(t)$, and the state unit $\boldsymbol{x}^{motor}(t)$ that induces a left turn.

After the second trial (Fig. S4B), if the robot touches the tactile cue on the left side, the state unit of the tactile module $\boldsymbol{x}^{tactile}(t)$ takes a positive value. The virtual unit of the tactile module $\tilde{\boldsymbol{x}}^{tactile}(t)$ is normally not activated because any correlations from the other modules to the tactile one are learned less often. Therefore, the difference of the virtual and state unit $\Delta\tilde{\boldsymbol{x}}^{tactile}(t)$ takes a negative value. By Eq. 3, this negative activity propagates through the learned connections to induce left turn.

These two neuronal paths have significant roles on solving the task. Additionally Bovet mentioned in his Ph.D. thesis [21] a connection that has a secondary role: the "tactile – vision" path can support the "reward – vision – motor" path. Any other connections are not mentioned in Bovet's paper, and they may not be necessary to solve the task. As described in the method and the result section, we cut those redundant connections, and proved that the robot was still able to solve the maze. However, we observed no VTE with this minimal connectivity, which let us to conclude the redundant connectivity is necessary for generating VTE.

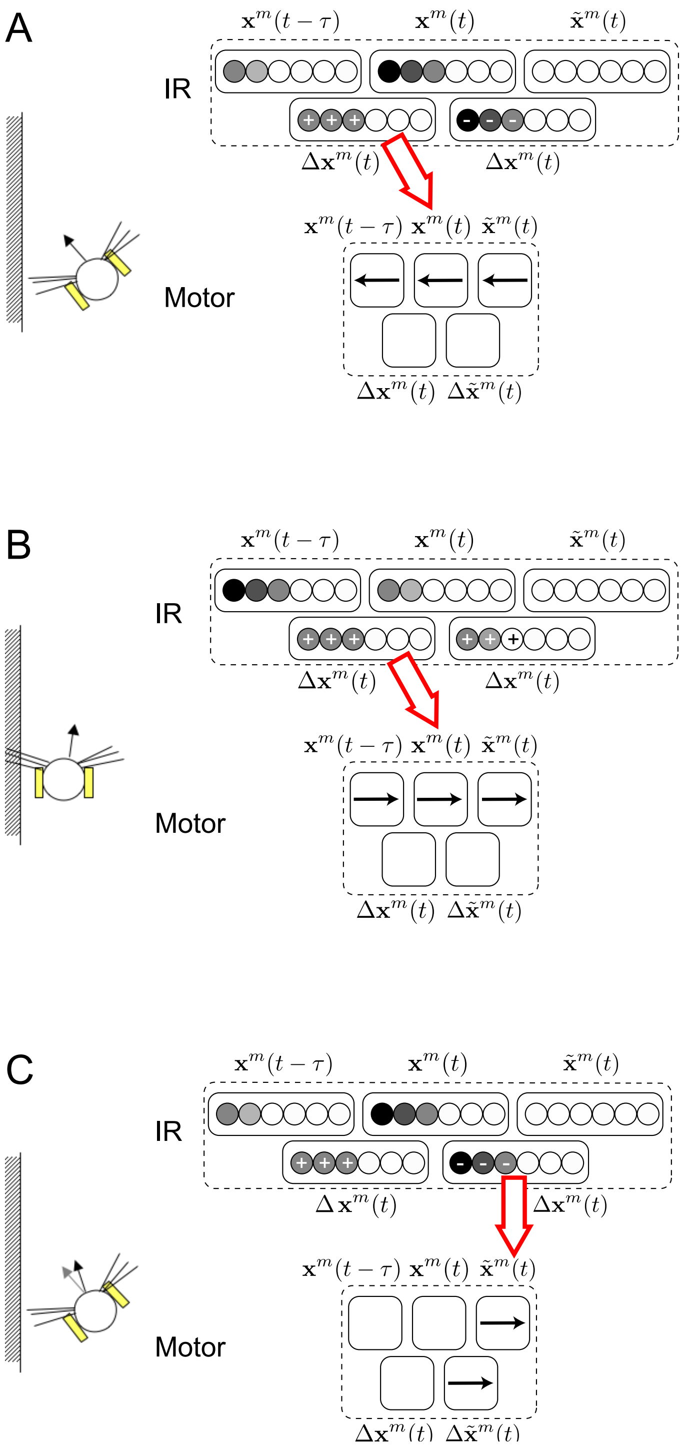

Figure S1. Illustration of the learning process of the "IR – motor" path during the training phase. This path lets the robot avoid the walls, and is learned in the training phase. Five boxes in each module show activities of each of the five types of variables used for computation of Eq. 2 and 3. The IR module has circles in each of the five boxes, which denote activity levels of each of the six IR neurons. In the motor module, the arrow indicates the direction of turning. A: When the robot turns to a wall, to the left wall for example, the robot learns a correlation between an increase of IR proximity signal in the left sensors and motor activity corresponding to turning to left. B: Conversely, it learns a correlation between a decrease of IR signal in the left sensors and motor activity corresponding to turning to right. C: After learning the correlation above, if it gets close to the left wall by making a left turn, the difference between the virtual and state units propagates into the virtual unit of the motor module, which generates an opposite turn (i.e., a right turn).

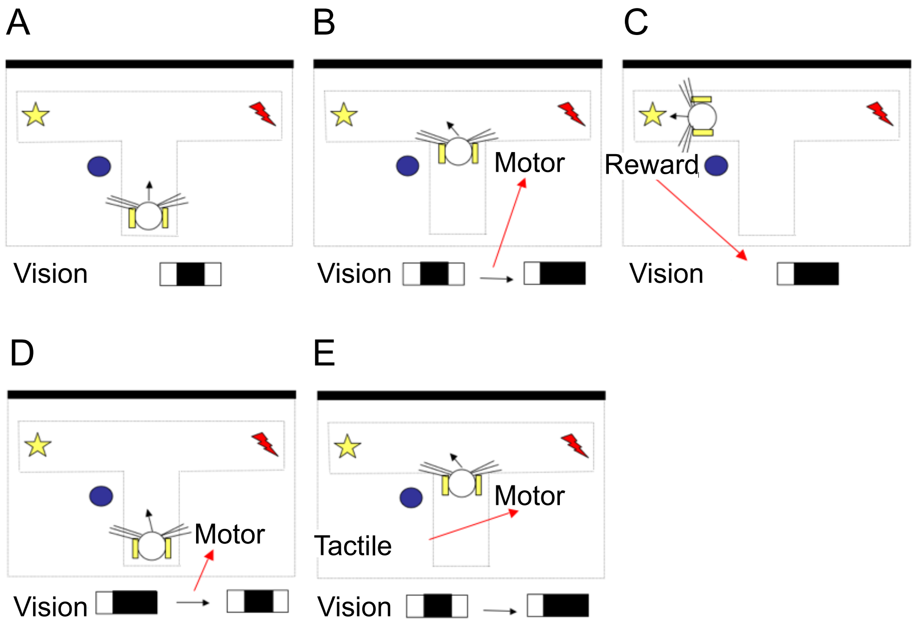

Figure S2. The process of learning. A, At the beginning of each trial, the robot is placed on the central arm of the T-maze. The camera detects the black wall at the back, and the central part of its visual field is activated. B, The robot has not get learned much correlations about the tactile cue so that the robot makes no large turn. But a slight difference in the right and left motors, because of asymmetry from the training phase, leads the robot to one side of the T-maze. C, When the robot reaches the reward, the Hebbian learning rule reinforces the correlation between an increase of reward and the camera image of the black wall. D, After getting the reward, the robot is returned to the starting position to start the second trial. Through the "reward – vision – motor" path learned at the first trial, the virtual unit of the reward module (always set to 1.0) propagates into the vision module, inducing a left turn, i.e., a turn to the previous reward position. E, During the turning, the left whiskers perceive the tactile cue, so that a "tactile – motor" correlation is produced.

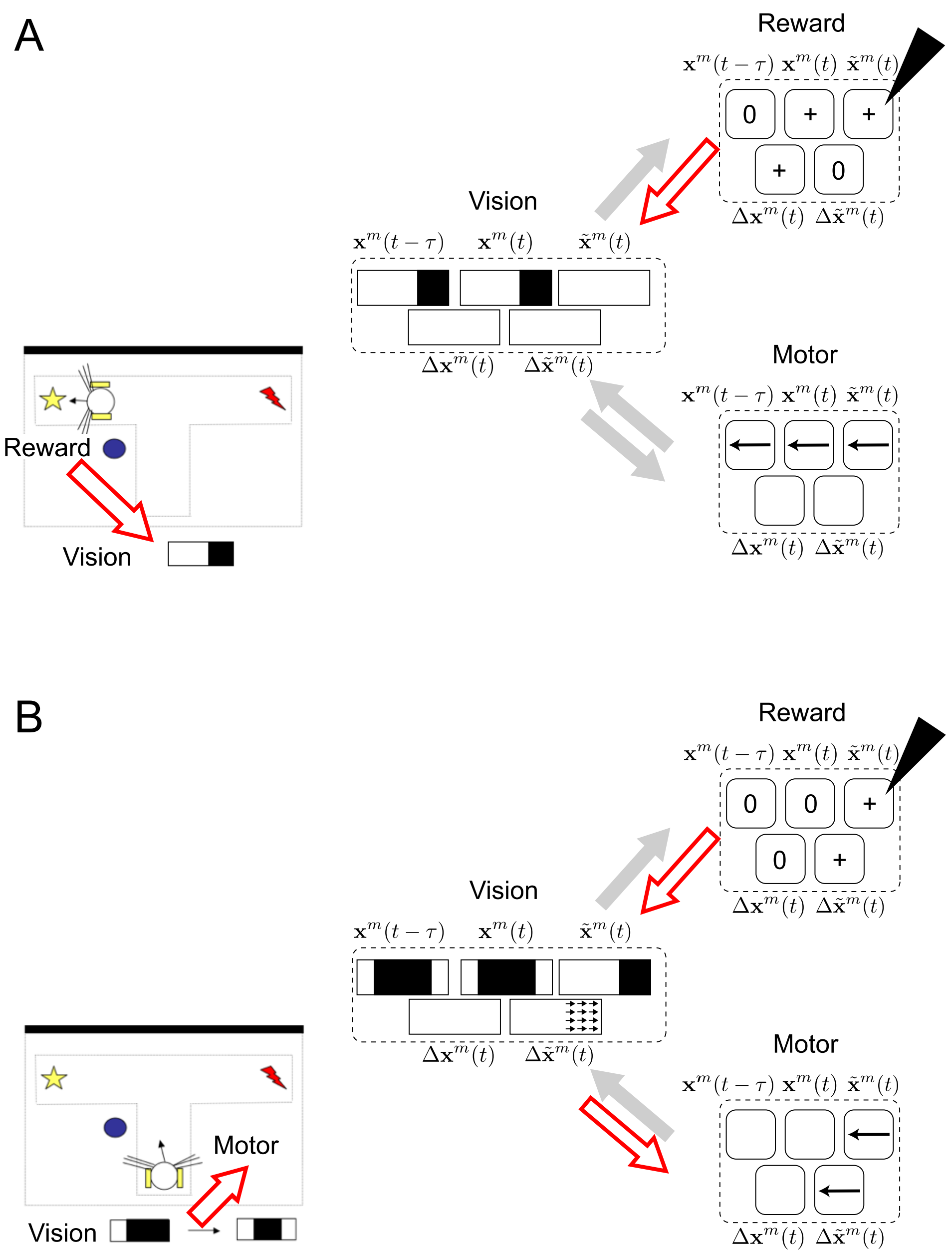

Figure S3. Illustration of the learning process of the "reward – vision – motor" path during the first and second trials. Five boxes in each module show activities of each of the five types of variables used for computation of Eq. 2 and 3. The five boxes inside the vision module denote activity patterns of its visual field (upper three boxes) or optical flow (lower two boxes). As for the reward module, the sign inside the boxes indicates its activity level. The virtual unit of the reward module $\tilde{\boldsymbol{x}}^{reward}(t)$ is always set to 1.0, which is illustrated by the black triangle. A, Neural activity when the robot gets the reward at the first trial. When it reaches the reward, the robot gets an increase of the reward signal (denoted by '+'), and at the same time the camera detects the black wall on the right side of its visual field. The robot learns a positive correlation between $\Delta\boldsymbol{x}^{reward}(t)$ and $\boldsymbol{x}^{vision}(t)$. B, Neural activity at the beginning of the second trial. By Eq. 3, the signal from the reward virtual unit $\boldsymbol{x}^{reward}(t)$ propagates through the synaptic connectivity to the vision module learned in A, to induce in the virtual unit of the vision module $\tilde{\boldsymbol{x}}^{vision}(t)$ a similar activity pattern of the camera image at the reward point in the first trial, i.e., the black wall image on the right part of its visual field. At the same time, the actual camera image (or the state unit $\boldsymbol{x}^{vision}(t)$) has its central visual field black. Therefore, the difference between them (or $\Delta\tilde{\boldsymbol{x}}^{vision}(t)$) has a visual flow in the right direction. This optical flow in the virtual unit propagates into the motor module to induce a left turn, because of the correlation learned in the training phase. Therefore, at the second trial, the robot is again successful.

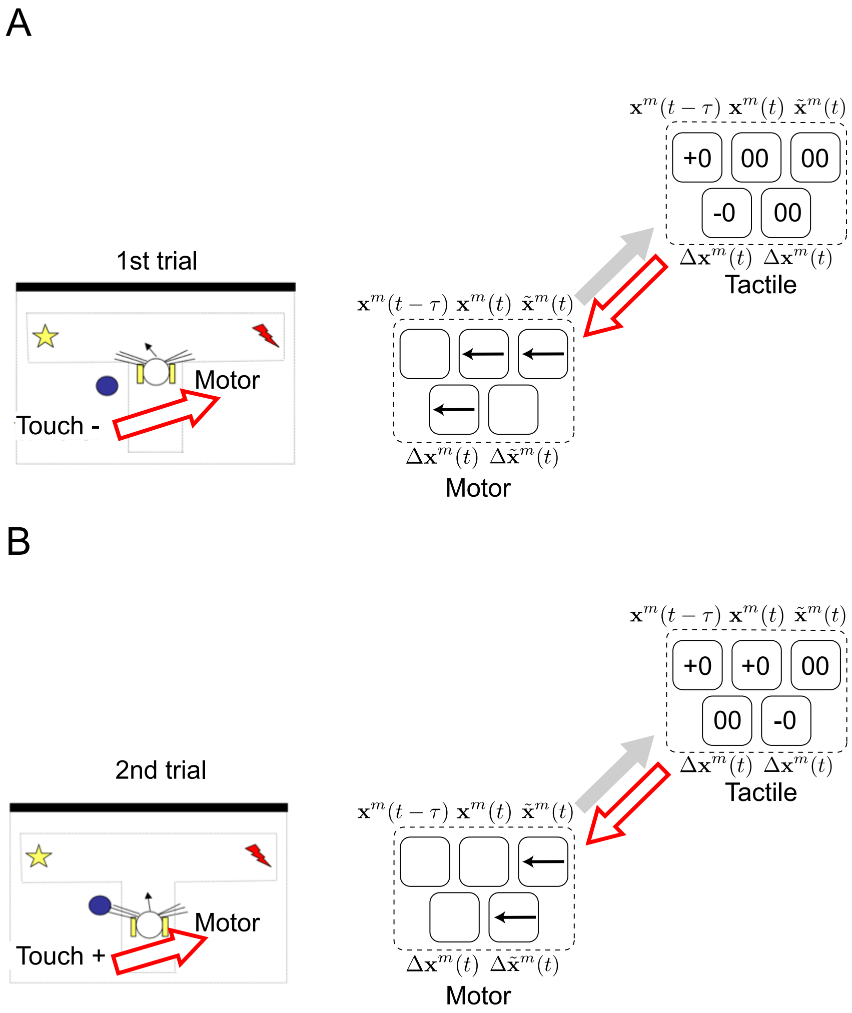

Figure S4. Illustration of the learning process of the "tactile – motor" path in the second trial. Five boxes in each module show activities of each of the five types of variables used for computation of Eq. 2 and 3. The sign in the boxes at the tactile module denotes reward(+), punishment(-) and nothing(0) for the left and right sides of the whiskers. A, Because of the "reward – vision – motor" path, the robot makes a left turn, and at the same time the left whiskers break contact with the tactile cue. Therefore, the robot learns a correlation between the decrease of the state unit $\Delta\boldsymbol{x}^{tactile}(t)$, and the state unit $\boldsymbol{x}^{motor}(t)$ that induces a left turn. B, After the second trial, if the robot touches the tactile cue on the left side, the state unit of the tactile module $\boldsymbol{x}^{tactile}(t)$ takes a positive value. The virtual unit of the tactile module $\tilde{\boldsymbol{x}}^{tactile}(t)$ is normally not activated because any correlations from the other modules to the tactile are not learned often. Therefore, the difference of the virtual and state unit $\Delta\tilde{\boldsymbol{x}}^{tactile}(t)$ takes a negative value. This negative activity propagates through the learned connections to induce a left turn.
